# Mitochondrial genome of the Spanish dancer sea slug *Hexabranchus sanguineus* (Nudibranchia) and its phylogenetic placement among dorids

**DOI:** 10.1080/23802359.2026.2669896

**Published:** 2026-05-19

**Authors:** Ye Zhu, Wenxin Zeng, Zhen Ni, Yuxin Wang, Ziming Su, Jiaqi Sun

**Affiliations:** a Yazhou Bay Innovation Institute/Hainan Key Laboratory for Coastal Marine Eco-environment and Carbon Sink/College of Ecology and Environment, Hainan Tropical Ocean University, Sanya, China; b College of Marine Biology and Fisheries, Hainan University, Haikou, China

**Keywords:** *Hexabranchus sanguineus*, mitochondrial genome, Nudibranchia, phylogeny, doridina

## Abstract

The complete mitochondrial genome of *Hexabranchus sanguineus* (Rüppell & Leuckart, 1828) is 14,550 bp and contains 13 protein-coding genes, 22 tRNAs, and two rRNAs. The genome is A + T-rich (63.2% in PCGs) with a strong bias toward A/U-ending codons, and exhibits a gene order highly conserved among dorid nudibranchs. Phylogenetic analyses consistently place *H. sanguineus* within Doridina, showing close affinity to Dorididae and Chromodorididae, while its unique morphological traits—including multiple gill tufts and active swimming—distinguish it from these relatives. These mitogenomic features support the taxonomic placement of *H. sanguineus* and provide a refined phylogenetic framework for dorid nudibranchs, reinforcing its value as a model for evolutionary studies in marine gastropods.

## Introduction

The molluscan clade Opisthobranchia comprises a morphologically diverse lineage of gastropods, defined by two pairs of tentacles and a postcardiac gill (Dinapoli and Klussmann-Kolb 2010). With over 6,000 described species distributed across global marine habitats, opisthobranchs have evolved diverse defensive adaptations, including chemical secretion, camouflage, and the sequestration of diet-derived bioactive compounds across multiple lineages (Dean and Prinsep 2017). Within Opisthobranchia, nudibranchs (sea slugs) represent a highly derived, often conspicuously pigmented subgroup. While adult nudibranchs are shell-less, most species retain a vestigial, transient shell during their planktonic larval stage (Clark 1975). This group is of significant research interest due to its association with bioactive secondary metabolites, many of which exhibit promising antitumor, antimicrobial, and antifouling activities (Dean and Prinsep 2017).

The family Hexabranchidae is monotypic, containing only the genus *Hexabranchus*, which currently includes two formally recognized species: *Hexabranchus morsomus* and the focal species of this study, *Hexabranchus sanguineus* (Rüppell & Leuckart 1828) (Valdés 2002). Commonly known as the Spanish dancer for its rhythmic undulatory swimming behavior, *H. sanguineus* is widely distributed across the Indo-Pacific region. It is morphologically characterized by vivid deep-red body coloration with white marginal markings, pale gills with white rachises, recurved mantle margins, and a maximum body length exceeding 40 cm, making it one of the largest known nudibranch species. It also exhibits atypical traits within the superfamily Eudoridoidea, including multiple gills inserting independently into the dorsal body wall and the absence of a protective gill pouch (Burn 2015; Tibiriçá et al. 2023).

Prior to experimental analyses, we confirmed the taxonomic identity of *H. sanguineus* specimens collected from coastal waters off Sanya, Hainan Island, China, using an integrated morphological and molecular approach. Cytochrome c oxidase subunit I (*cox1*) barcode sequences from the assembled mitochondrial genome showed 99.09% identity with the validated *H. sanguineus* reference sequence in NCBI GenBank (Supplementary Figure 1), and 99.7% identity with the corresponding reference in the Barcode of Life Data (BOLD) System (Supplementary Figure 2), ruling out cryptic species or congeneric misidentification. Morphological examination confirmed all diagnostic synapomorphies of Hexabranchidae and *H. sanguineus*, consistent with the original species description and recent taxonomic revisions (Tibiriçá et al. 2023).

This study presents the complete mitochondrial genome of *H. sanguineus*, characterizes its genomic features, and resolves its phylogenetic placement within Nudibranchia. Our findings synthesize existing data on the species’ systematics and biology, while further validating its utility as a model organism for investigating marine evolutionary adaptations.

## Materials and methods

**Figure 1.**
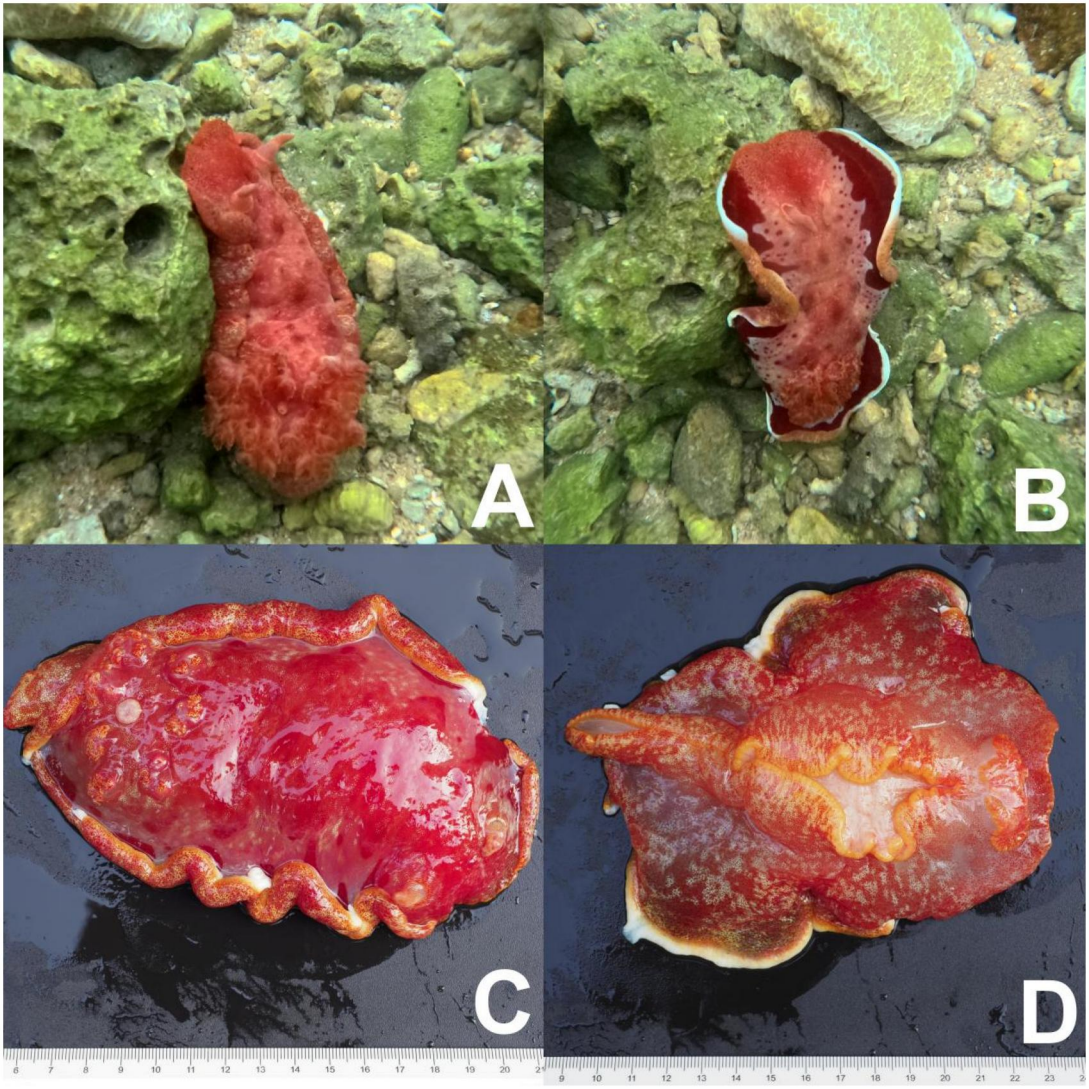
*Hexabranchus sanguineus* is a large, colorful sea slug characterized by a mottled red coloration with white, orange, and pink hues. It exhibits six exposed gills and a fleshy foot covered with numerous microscopic hairs, and can attain lengths of up to 40 cm. (a) Still image of *Hexabranchus sanguineus* in its habitat; (b) motion shot of *Hexabranchus sanguineus* in its habitat; (c) dorsal view; (d) ventral view.

One *H. sanguineus* specimen was collected in April 2025 from coastal waters off Wuzhizhou Island, Sanya, Hainan Province, China (18°18′02.118″N, 109°45′27.913″E). Morphological identification was based on diagnostic traits, including a bright-red body with white marginal bands and low-profile tubercles across the dorsum (Figure 1). The voucher specimen (accession No. 01R0561) is preserved in 99.9% (v/v) ethanol at the Science and Technology Center Building, No. 3 Shanghai Road, Qingdao, China (corresponding contact: Jinhu Mu, mujh@biomarker.com.cn).

Total genomic DNA was extracted from dissected muscle tissue using the TIANamp Genomic DNA Kit (TIANGEN, Beijing, China) following the manufacturer’s protocol. Sequencing libraries were constructed with the TIANSeq Fast DNA Library Kit (average insert size 350 bp), and paired-end sequencing (2 × 150 bp) was performed on the Illumina HiSeq 2000 platform. Raw reads were processed using Fastp (Chen et al. 2018) to trim adapters and filter low-quality bases, yielding 29,747,075 high-quality clean paired-end reads. The maximum, average, and minimum mitochondrial genome coverage were calculated as 559×, 333.16×, and 224×, respectively, using SAMtools v1.7 (Li et al. 2009) (Supplementary Figure 3). *De novo* assembly of the complete mitochondrial genome was performed using GetOrganelle, followed by manual inspection and circularization of the full mitogenome contig. Gene annotation, including protein-coding genes (PCGs), ribosomal RNA (rRNA) genes, and transfer RNA (tRNA) genes, was conducted using MITOS2 (Donath et al. 2019) and was subsequently subjected to manual curation for all annotated features. A graphical map of the annotated mitogenome was generated using OGDraw (Greiner et al. 2019).

For phylogenetic analysis, mitogenome sequences from 23 closely related nudibranch species (representing Chromodorididae, Dorididae, Hexabranchidae, Volvatellidae, and Cadlinidae) were retrieved from GenBank, with Runcina aurata designated as the outgroup (24 total taxa). Conserved mitochondrial PCGs across all taxa were extracted using PhyloSuite v1.2.2 (Zhang et al. 2020). Each PCG was aligned using MAFFT v7.313 (Katoh and Standley 2013), and the resulting alignments were concatenated using PhyloSuite’s built-in function with default trimming parameters.

To identify the optimal partitioning scheme and nucleotide substitution models for phylogenetic inference, we utilized two complementary analytical approaches. For maximum likelihood (ML) analysis, ModelFinder (Kalyaanamoorthy et al. 2017) (implemented within IQ-TREE) was used to select the best-fit partitioning scheme and nucleotide substitution models based on the Bayesian Information Criterion (BIC). For nucleotide datasets, the general time-reversible (GTR) model with empirical base frequencies (F), free-rate heterogeneity (R), and/or invariant sites (I) was consistently identified as the optimal model. For amino acid datasets (used to mitigate phylogenetic saturation effects), the mitochondrial-specific substitution matrix mtMet was applied. ML phylogenetic inference was performed using IQ-TREE (Nguyen et al. 2015) under an edge-unlinked partitioning scheme, with nodal support values estimated *via* 5,000 ultrafast bootstrap replicates. For Bayesian inference (BI) analysis, PartitionFinder 2 (Lanfear et al. 2017) was used to determine the best-fitting substitution models under the Akaike Information Criterion (AIC). The GTR model with invariant sites and gamma-distributed rate heterogeneity (GTR+I + Γ) was identified as optimal for the majority of nucleotide partitions, while the JTT model with + I + G + F parameters was selected for amino acid alignments. BI phylogenetic reconstruction was conducted using MrBayes v3.2.6 (Ronquist et al. 2012) on the CIPRES Science Gateway, with two independent Markov chain Monte Carlo (MCMC) runs performed in parallel. Each run comprised four chains (three heated and one cold) and was executed for 10 million generations, with sampling occurring every 1,000 generations. Convergence of the runs was verified by confirming that the average standard deviation of split frequencies fell below 0.01. The first 25% of sampled trees were discarded as burn-in, and a 50% majority-rule consensus tree was generated to summarize posterior probability values for all nodes.

## Results

**Figure 2.**
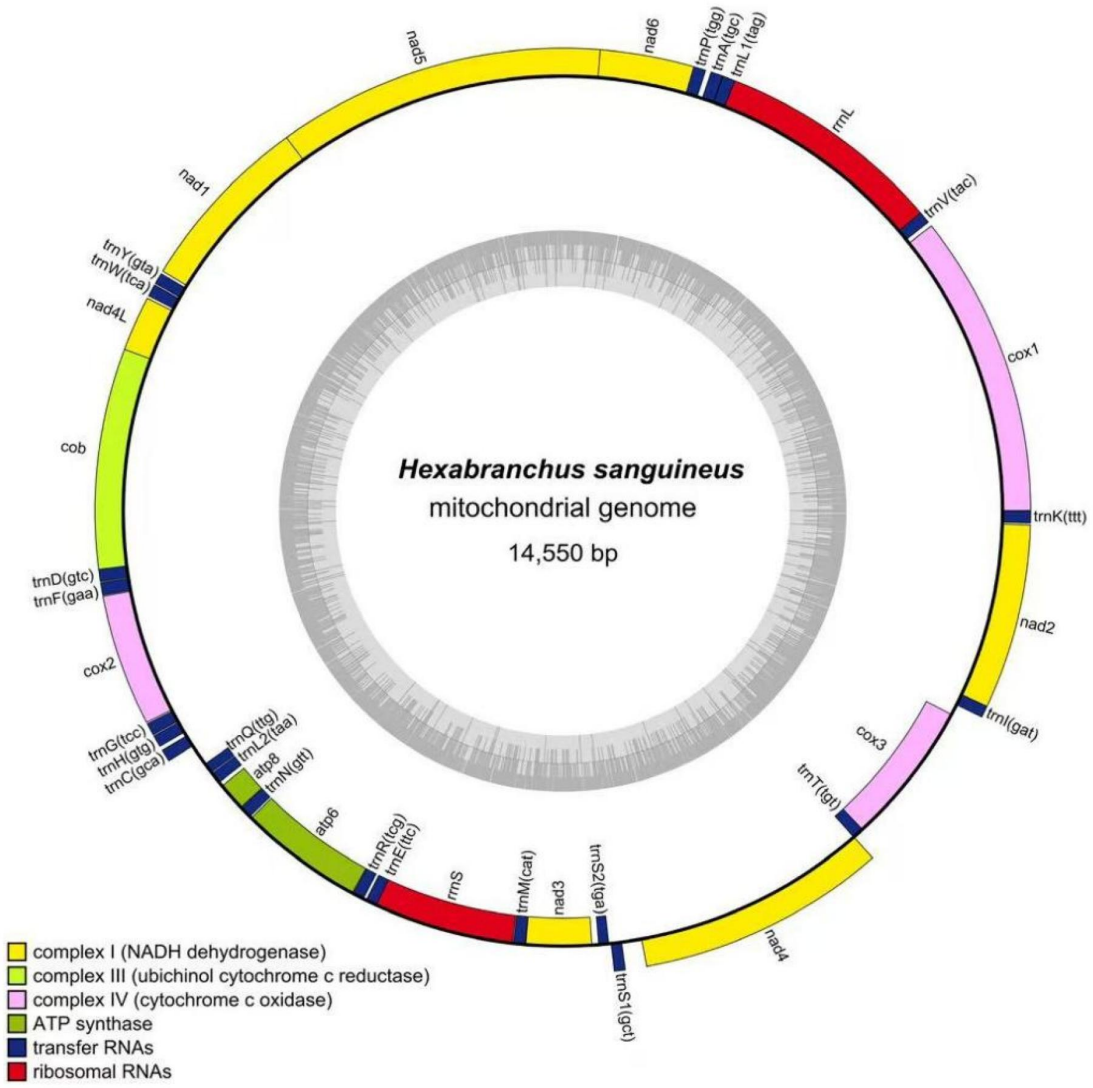
The structural map of the mitochondrial genome of *Hexabranchus sanguineus.*

The complete circular mitochondrial genome of *H. sanguineus* is 14,550 bp in length, encoding 37 genes typical of metazoan mitogenomes: 13 PCGs, 22 tRNA genes, and 2 rRNA genes. The annotated sequence has been deposited in NCBI GenBank under accession number PX421547. The overall nucleotide composition is 27.88% adenine (A), 20.50% guanine (G), 36.66% thymine (T), and 14.96% cytosine (C), with an AT skew of −0.136 and GC skew of 0.156 (Figure 2).

The 13 PCGs have a combined length of 10,902 bp, with an overall A + T content of 63.2%. Six PCGs (*cox1*, *nad5*, *nad1*, *nad4*, *cox3*, *nad2*) use the TAG stop codon, while the remaining seven terminate with TAA. Four PCGs (*cox3*, *atp6*, *atp8*, *nad3*) are encoded on the light (L) strand, with the other nine encoded on the heavy (H) strand. The two rRNA genes, 16S rRNA (*rrnL*, 1,184 bp) and 12S rRNA (*rrnS*, 755 bp), have A + T contents of 68.2% and 67.1%, respectively; *rrnL* is encoded on the H strand, and *rrnS* on the L strand. The 22 tRNA genes have a total length of 1,434 bp (overall A + T content 65.6%), with the majority encoded on the H strand.

Phylogenetic trees reconstructed using ML and BI methods based on the concatenated PCG dataset showed largely congruent topologies, with high support for most nodes. All taxa belong to the class Gastropoda, with two well-resolved major clades recovered in both analyses. The first clade comprised *Runcina aurata* and *Ascobulla fragilis* (order Runcinacea), forming the basal outgroup. All remaining taxa (order Nudibranchia) formed a strongly supported monophyletic group (bootstrap support = 99.5% in ML; PP = 1.0 in BI). *H. sanguineus*, the sole representative of Hexabranchidae, was nested within the Nudibranchia clade, with a distant phylogenetic relationship to the basal Runcinacea lineage.

**Figure 3.**
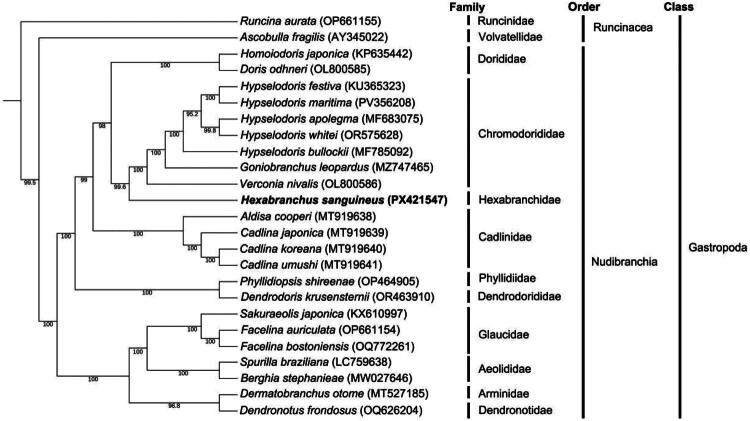
** ** A maximum-likelihood phylogenetic tree was constructed based on the protein-coding genes on the mitochondrial genome of *Hexabranchus sanguineus* (PX421547) and 23 related species, with *Runcina aurata* OP661155 (Galià-Camps et al. 2024) used as the outgroup. Numbers on nodes indicate ML bootstrap values (%). the following sequences were used: AY345022 (Unpublished), KP635442 (Liu et al. 2016), OL800585(Unpublished), KU365323 (Karagozlu et al. 2016), PV356208 (Unpublished), MF683075 (Unpublished), OR575628 (Unpublished), MF785092 (Unpublished), MZ747465 (Unpublished), OL800586 (Unpublished), PX421547 (this study), MT919638 (Unpublished), MT919639 (Unpublished), MT919640 (Unpublished), MT919641 (Unpublished), OP464905 (Unpublished), OR463910 (lee et al. 2024), KX610997 (Karagozlu et al. 2016), OP661154 (Galià-Camps et al. 2024), OQ772261 (Unpublished), LC759638 (mizobata et al. 2023), MW027646 (Melo Clavijo et al. 2021), MT527185 (Unpublished), OQ626204 (Unpublished).

Minor topological differences were observed between the ML and BI trees in the fine-scale placement of *H. sanguineus*. In the ML tree, *H. sanguineus* formed a well-supported sister clade with *Verconia nivalis* (Chromodorididae; bootstrap support = 99.6%), which was successive sister to a clade containing *Goniobranchus leopardus* and multiple *Hypselodoris* species (Chromodorididae; all relevant node support >95%). This broader clade formed a highly supported subclade with Cadlinidae representatives (bootstrap support = 99%), which was sister to a second major Nudibranchia clade containing Glaucidae, Aeolidiidae, Arminidae, and Dendronotidae (bootstrap support = 100%) (Figure 3).

**Figure 4.**
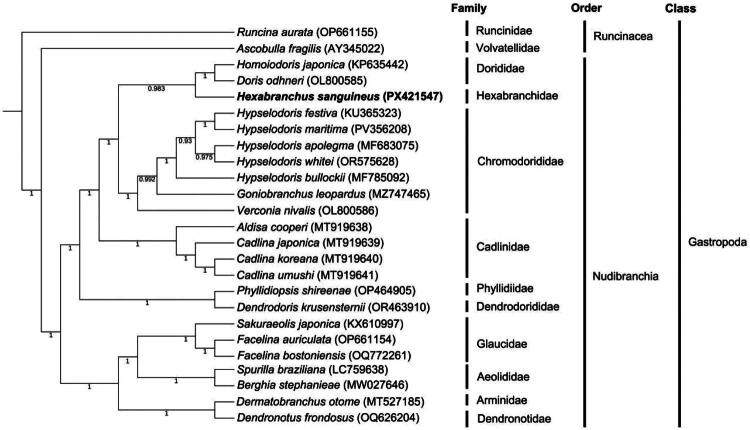
** ** A Bayesian inference (BI) phylogenetic tree was constructed based on the protein-coding genes on the mitochondrial genome of *Hexabranchus sanguineus* (PX421547) and 23 related species, with *Runcina aurata* OP661155 (Galià-Camps et al. 2024) used as the outgroup. The analysis was conducted using MrBayes v3.2.6 under the GTR+I + Γ nucleotide substitution model. The numbers at the nodes represent posterior probabilities (PP). The following sequences were used: AY345022 (Unpublished), KP635442 (Liu et al. 2016), PX421547 (this study), OL800585(Unpublished), KU365323 (Karagozlu et al. 2016). PV356208 (Unpublished), MF683075 (Unpublished), OR575628 (Unpublished), MF785092 (Unpublished), MZ747465 (Unpublished), OL800586 (Unpublished), MT919638 (Unpublished), MT919639 (Unpublished), MT919640 (Unpublished), MT919641 (Unpublished), OP464905 (Unpublished), OR463910 (lee et al. 2024), KX610997 (Karagozlu et al. 2016), OP661154 (Galià-Camps et al. 2024), OQ772261 (Unpublished), LC759638 (mizobata et al. 2023), MW027646 (Melo Clavijo et al. 2021), MT527185 (Unpublished), OQ626204 (Unpublished).

In the BI tree, *H. sanguineus* clustered with *Homoiodoris japonica* and *Doris odhneri* (Dorididae;PP = 0.983), with this clade sister to a clade of *Hypselodoris*, *Goniobranchus*, and *Verconia* species (Chromodorididae; all relevant node PP >0.93). Consistent with the ML tree, the majorclade containing *H. sanguineus* formed a maximally supported subclade with Cadlinidae, Phyllidiidae, and Dendrodorididae (PP = 1.0), which was sister to the clade of Glaucidae, Aeolidiidae, Arminidac, and Dendronotidae (PP = 1.0). In both phylogenies, *H. sanguineus* was placed in a distinct major clade separate from the Glaucidac-Aeolidiidae-Arminidae-Dendronotidae lineage, indicating a relatively distant phylogenetic relationship between these groups (Figure 4).

## Discussion and conclusion

The 14.55 kb circular mitochondrial genome of *H. sanguineus* encodes 37 standard metazoan mitochondrial genes, a composition and structural organization consistent with the typical profile of dorid nudibranchs and opisthobranch mollusks, including previously published mitogenomes of *V. nivalis* and *D. odhneri*. Mitochondrial genes are distributed across both heavy and light strands, matching the pattern observed in other opisthobranch lineages. The genome exhibits a strongly A + T-rich nucleotide composition (∼63% total A + T content), with a slight negative AT skew and positive GC skew. These skew values are highly congruent with those reported for other dorid nudibranchs, including *V. nivalis* (AT skew ≈ −0.167) and *D. odhneri* (AT skew=-0.089), confirming that *H. sanguineus* shares the characteristic AT nucleotide bias widely observed in nudibranch mitogenomes (Do et al. 2022; Li et al. 2025).

Consistent with its AT-rich composition, the *H. sanguineus* mitogenome shows a strong bias toward A/U-ending codons, as demonstrated by relative synonymous codon usage (RSCU) analysis. This pattern aligns with that documented in other dorid nudibranchs, with A/U-ending codons highly represented and G/C-ending codons consistently underrepresented across nudibranch mitogenomes (Li et al. 2025). UUA (encoding Leucine), the most abundant codon in the *H. sanguineus* mitogenome, is also the most frequently used codon in closely related dorid species, reflecting conserved AT-rich mutational pressure acting on these mitogenomes. *V. nivalis*, *H. japonica*, and *D. odhneri* exhibit highly similar codon usage profiles to *H. sanguineus*, with most preferred codons terminating in A/U (Do et al. 2022). Pairwise comparisons revealed *D. odhneri* has the most similar codon usage pattern to *H. sanguineus*, while *H. japonica* is the most divergent. The CGG codon (encoding Arginine) was a consistent outlier across all comparisons, indicating highly variable arginine codon usage across these nudibranch mitogenomes, with potential utility as a lineage-specific molecular marker for dorid nudibranchs (Liu et al. 2016).

Mitochondrial gene organization of *H. sanguineus* is highly conserved relative to close dorid relatives: when linearized using *cox1* as the reference anchor, all 13 PCGs and both rRNA genes show identical gene order across *H. sanguineus*, *V. nivalis*, *D. odhneri*, and *H. japonica*. This aligns with previous reports of highly conserved gene order in dorid nudibranchs consistent with the consensus nudibranch mitogenome arrangement, with no large-scale gene rearrangements detected in the *H. sanguineus* mitogenome (Do et al. 2022; Li et al. 2025). While rare gene rearrangements have been documented in other opisthobranch lineages, the *H. sanguineus* mitogenome adheres strictly to the consensus dorid mitogenome architecture.

Phylogenetic analyses using both BI and ML consistently resolved *H. sanguineus* (Hexabranchidae) as a distinct, well-supported lineage with close phylogenetic affinity to the dorid families Dorididae and Chromodorididae, with minor topological variation between the two reconstructions: the BI tree resolved *H. sanguineus* as the direct sister group to Dorididae, while the ML tree placed it as sister to a clade uniting Dorididae and Chromodorididae. These three families share core morphological and ecological traits diagnostic of dorid nudibranchs, including a dorsoventrally flattened body plan, specialized rhinophores, dorsally positioned gills, a benthic spongivorous diet, and the ability to sequester diet-derived chemical defenses (Burn 2015). However, *H. sanguineus* exhibits unique derived apomorphies, including multiple independently contractile gill tufts, active swimming *via* rhythmic mantle undulations, exceptional body size, and a pronounced startle response—traits that distinguish it from smaller, typically aposematic Chromodorididae and cryptic Dorididae species (Valdés 2002; Tibiriçá et al. 2023).

In summary, the mitogenomic features of *H. sanguineus* strongly support its taxonomic placement within the suborder Doridina, with a particularly close genetic affinity to the Dorididae lineage. These findings complement and refine existing phylogenetic frameworks for dorid nudibranchs, further establishing *H. sanguineus* as a valuable model for evolutionary studies in this diverse gastropod group.

## Supplementary Material

Supplementary Figure 2.jpg

Supplementary Figure 3.jpg

Supplementary Figure 1.jpg

## Data Availability

The genome sequence data that support the findings of this study are openly available in GenBank of NCBI at https://www.ncbi.nlm.nih.gov/, under the accession no. PX421547. The associated BioProject, SRA, and BioSample numbers are PRJNA1357818, SRR35949769, and SAMN53088618 respectively.
