# Supplementary figures and images for: Mitochondrial genome of the Spanish dancer sea slug Hexabranchus sanguineus (Nudibranchia) and its phylogenetic placement among dorids
Source: Mitochondrial DNA B Resour. 2026 May 19;11(6):762–8. doi: 10.1080/23802359.2026.2669896 (PMC13188574; doi:10.1080/23802359.2026.2669896)

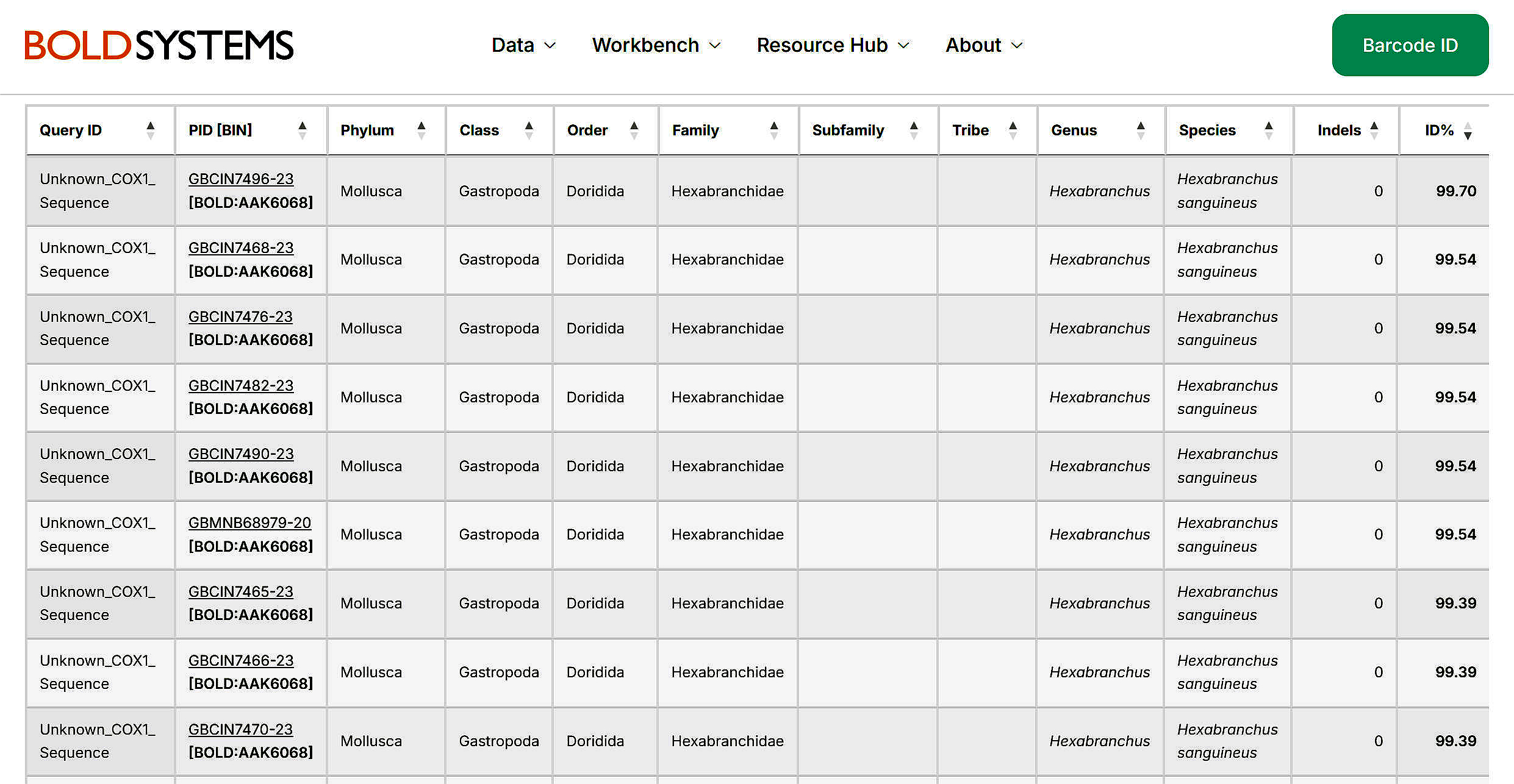

Supplement: Supplementary Figure 2.jpg [file TMDN_A_2669896_SM0294.jpg]

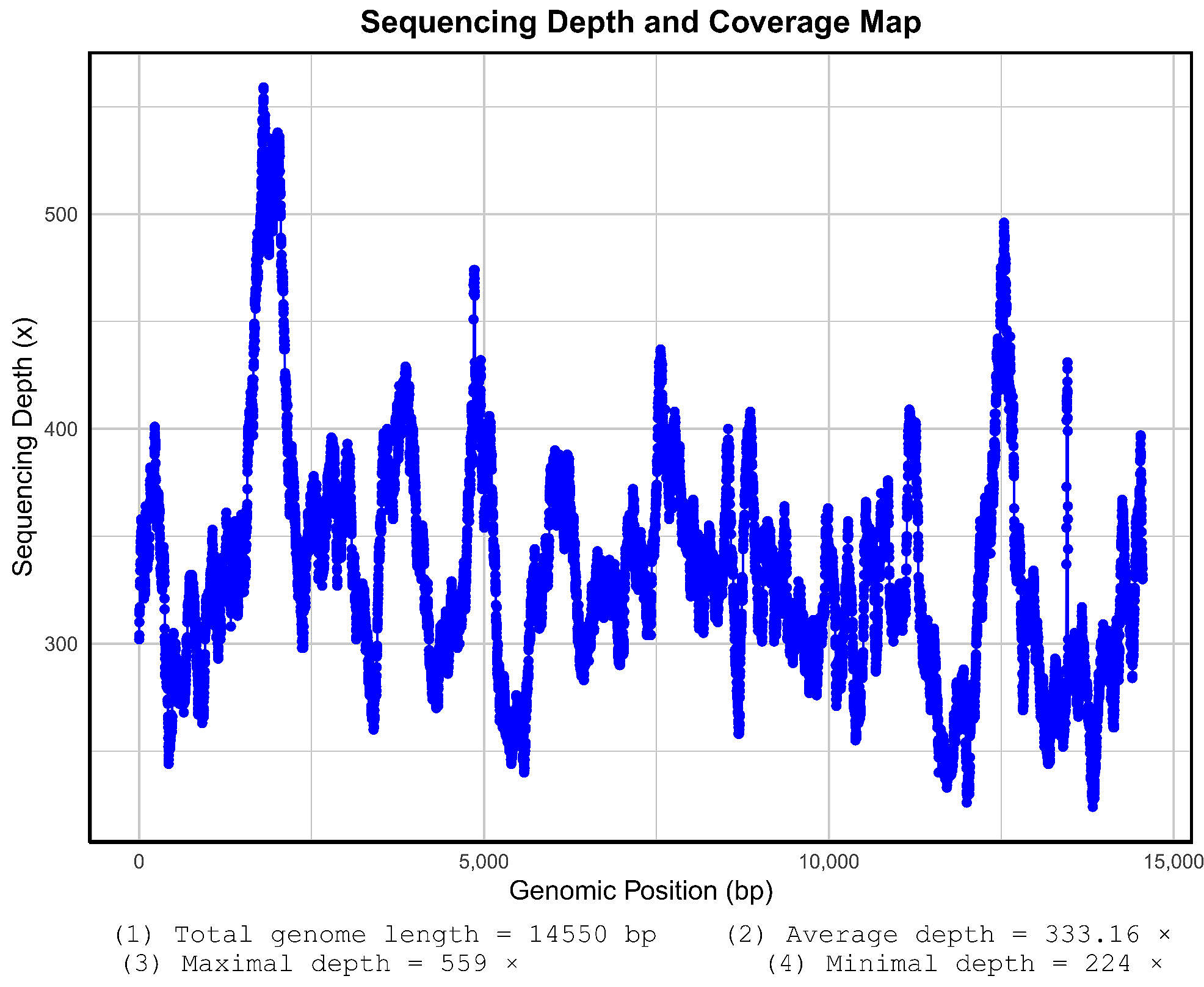

Supplement: Supplementary Figure 3.jpg [file TMDN_A_2669896_SM0291.jpg]

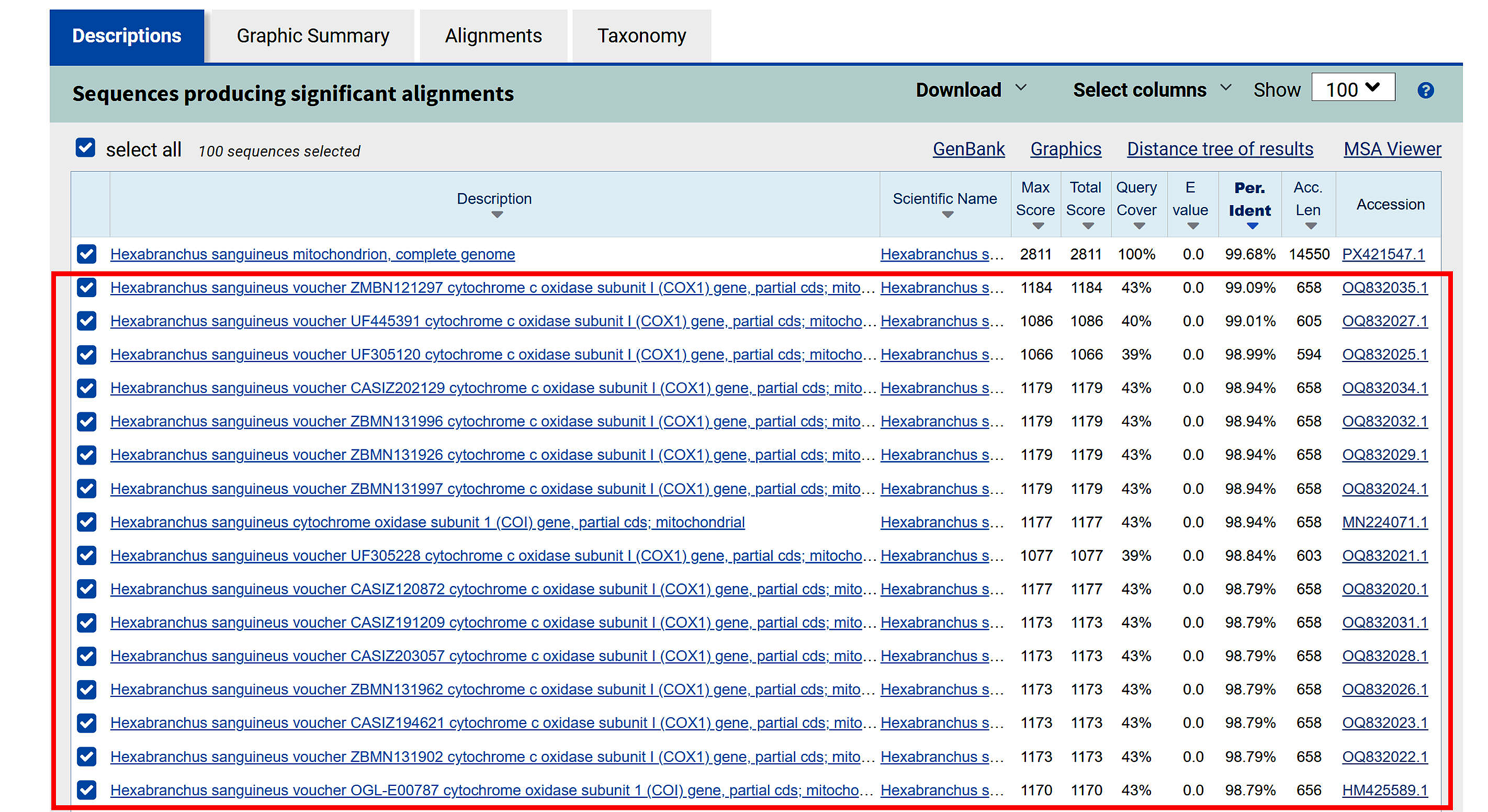

Supplement: Supplementary Figure 1.jpg [file TMDN_A_2669896_SM0289.jpg]
